# Network Pharmacology Approach for Medicinal Plants: Review and Assessment

**DOI:** 10.3390/ph15050572

**Published:** 2022-05-04

**Authors:** Fatima Noor, Muhammad Tahir ul Qamar, Usman Ali Ashfaq, Aqel Albutti, Ameen S. S. Alwashmi, Mohammad Abdullah Aljasir

**Affiliations:** 1 Department of Bioinformatics and Biotechnology, Government College University Faisalabad, Faisalabad 38000, Pakistan; fatimanoor1122@yahoo.com (F.N.); tahirulqamar@gcuf.edu.pk (M.T.u.Q.); 2 Department of Medical Biotechnology, College of Applied Medical Sciences, Qassim University, Buraydah 51452, Saudi Arabia; 3 Department of Medical Laboratories, College of Applied Medical Sciences, Qassim University, Buraydah 51452, Saudi Arabia; aswshmy@qu.edu.sa (A.S.S.A.); mjasr@qu.edu.sa (M.A.A.)

**Keywords:** network pharmacology, medicinal plants, active ingredients, system biology, drug discovery

## Abstract

Natural products have played a critical role in medicine due to their ability to bind and modulate cellular targets involved in disease. Medicinal plants hold a variety of bioactive scaffolds for the treatment of multiple disorders. The less adverse effects, affordability, and easy accessibility highlight their potential in traditional remedies. Identifying pharmacological targets from active ingredients of medicinal plants has become a hot topic for biomedical research to generate innovative therapies. By developing an unprecedented opportunity for the systematic investigation of traditional medicines, network pharmacology is evolving as a systematic paradigm and becoming a frontier research field of drug discovery and development. The advancement of network pharmacology has opened up new avenues for understanding the complex bioactive components found in various medicinal plants. This study is attributed to a comprehensive summary of network pharmacology based on current research, highlighting various active ingredients, related techniques/tools/databases, and drug discovery and development applications. Moreover, this study would serve as a protocol for discovering novel compounds to explore the full range of biological potential of traditionally used plants. We have attempted to cover this vast topic in the review form. We hope it will serve as a significant pioneer for researchers working with medicinal plants by employing network pharmacology approaches.

## 1. Introduction

It has become a need of the hour to tackle the major concerns that the world has been confronted with regarding global health challenges [1]. Complex diseases, including cancer, diabetes, etc., draw researchers’ attention because these diseases are frequently caused by a malfunction of a complete regulatory network rather than a mutation or malfunctioning of a single gene [2]. As a result, the goal of diagnosing and treating complicated disorders may not be achieved by simply targeting a single gene. However, there is an urgent need to develop innovative approaches to target the entire biological networks that underlie the disease [3,4]. Thus, understanding the molecular pathways that govern disease prognosis are critical in the fight against complicated diseases [5].

**Figure 1:**
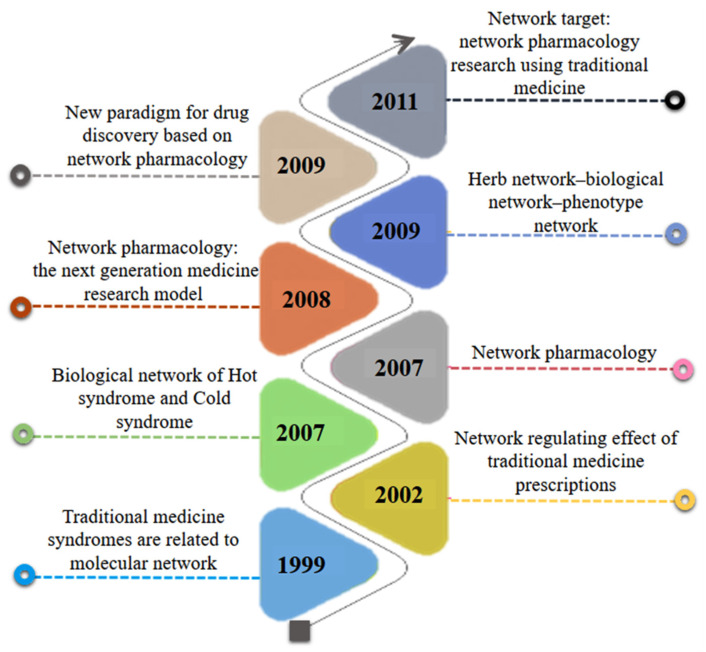
Timeline diagram representing the origin of network pharmacology.

Presently, natural products comprise a large portion of current-day pharmaceutical agents, most notably in the area of disease treatments [6]. Natural products have long been a huge storehouse of potent resources for mankind [7]. High throughput techniques have proposed a strong arm in screening the pharmacological efficacy of herbal medicines in drug discovery [8]. One unique way to learn more about how active substances perform their therapeutic effect is to predict the gene networks that are being regulated by active compounds of medicinal plants [9]. Drug discovery faces an efficacy crisis to which ineffective, mainly single-target, and symptom-based rather than mechanistic approaches have contributed. Current one drug–one target–one disease approaches in drug discovery have become increasingly inefficient. While single-target strategies might prove a useful approach for single gene disorders, however, for complicated diseases that are caused by the interaction of multiple genes, such one single-target approaches are not fruitful [10]. The concept of developing multi-target drugs against complex diseases such as diabetes and cancer is fast growing in drug discovery. Regarding this, network pharmacology defines disease mechanisms as networks best targeted by multiple, synergistic drugs. The use of network pharmacology to better understand the mechanism of action of herbal medicines has recently become popular [11,12]. In 2007, Hopkins coined the term “network pharmacology”, which is based on the idea that several highly efficient drugs act on numerous targets rather than just a single one [13]. Moreover, Figure 1 illustrates the origin of network pharmacology.

Network pharmacology is evolving as a frontier in drug discovery and development as it integrates systematic medicine with information science [14]. In the beginning, network pharmacology had a vague conception about drug discovery, and perhaps some overhyping of its promises, as found in the early stages of almost all new technologies [15,16]. At present, one can say that network pharmacology has begun to grow and is a commonly used approach in this modern era-drug discovery process [17]. Network pharmacology is an integrative in silico approach for establishing a “protein–compound/disease–gene” network to reveal the mechanisms underlying the synergistic therapeutic actions of traditional medicines [18]. This advancement, in turn, has shifted the paradigm from a “one-target, one-drug” mode to a “network-target, multiple-component-therapeutics” mode.

The use of bioactive compounds to reform medicines in the future is exciting, and prospects for curing multiple diseases are encouraging [19,20]. This review sheds light on potential correlations between target genes and active ingredients of medicinal plants from a network pharmacology perspective. The present study addresses the break in literature by presenting an integrated approach exploring drug–target interactions to better identify the novel inhibitors for a particular target and their mode of action. This literature review provides a comprehensive overview of the methodology, significance, and application of network pharmacology to cure a wide spectrum of complex diseases. To our knowledge, this baseline review aids researchers in understanding many aspects of biomedicine, from protein synthesis to health and diseases.

## 2. Network Biology to Network Pharmacology

The advent of highly efficient technologies for analyzing big data has opened new avenues for discovering more intriguing and effective diagnostic and therapeutic solutions [5,21]. Understanding how well the proteins interfere with the functioning of the complex regulatory machinery is critical [22]. This sparked the development of network biology, which asserts that biological networks are commanded by general principles that propose a unique theoretical foundation that ultimately changes our understanding regarding the biology of diseases. Numerous techniques for the construction of regulatory networks were proposed in the 21th century that used computational tools, particularly data mining, to explore the relationship among phenotypes and genotypes of diseases [23]. Innovations in network biology have revealed that single-protein targets are ineffective in treating complicated disorders [24,25]. This prompted drug developers to understand the principle of polypharmacology, which they had previously viewed as an ineffective approach that needed to be eradicated to develop a viable multi-target drug [26,27]. With the emergence of network pharmacology as a completely independent technique, a dramatic change has been observed from extremely specialized single-target drugs to multi-targeted drugs. The next era witnessed the integration of system biology with polypharmacology in various health sides [28,29,30].

## 3. Network Pharmacology and Traditional Medicine

Over the past decade, local communities used medicinal plants without scientific studies [31,32]. Various medicinal plant species have been utilized in traditional medicines [33,34]. Although medicinal plants impact people’s lives by providing low cost and natural remedies, the unsustainable use and traditional collection and application methods have resulted in the depletion of several plant species of precious worth [35,36]. Traditional medicines, which are described by holistic philosophy and extensive experimentation in multicomponent treatments, provide promising potential for controlling the complicated nature of disorders [37,38,39]. Using herbal formulae is a unique aspect of traditional medicine [40]. In this era of big data, the reengineering of traditional medicines may be performed by simply understanding the combinatorial nature of herbal formulae as well as their mechanisms of action [41,42]. Today’s network pharmacology provides a novel opportunity to investigate not only the molecular complexity of herbal formula but also the correlation that exist among the herbal formula and complicated disorders in a systematic manner [43,44]. Herbs used in traditional medicines have indicated a best molecular match, which might elicit a more consistent network reaction than a single drug [45,46,47]. Network-based methodologies are becoming more popular research tools in areas of new drug development. They assist in comprehending innovative treatments by utilizing natural products as the lead compound responsible for drug synergism and cumulative activity. These techniques have been proven to work in a variety of herbal compositions used in traditional medicine [48,49,50].

Network pharmacology is considered a modern-era approach for identifying active compounds and putative molecular targets from a wide variety of herbal formulae or simple herbs [51,52,53]. This integrated approach is a touchstone for the initial screening of medicinal plants’ bioactive compounds and a new therapeutic concept for further exploration on mechanisms’ active compounds for disease treatment [54,55]. As a result, incorporating network pharmacology in traditional medicine will offer unique and novel options for uncovering active compounds, biomarkers, and the scientific basis of traditional medicine based on the complicated biological systems of the human body [54,56,57].

## 4. From Polypharmacology to Network Pharmacology: The Need to Reengineer Botanical Drugs

Even if healing is magical, it is unbelievable that there is a therapeutic effect without molecular interactions between the biological target and treatment. Medicinal plants that hold millions of potentially active ingredients frequently fail to disclose the expected molecular mechanisms of action [3,58,59,60]. Usually, standard biochemical methods are incapable of elucidating viable action modes. Despite such an average response, more research is pressingly demanding steps to achieve outstanding output in the future. Various active ingredients in medicinal plants have no more-robust connections with target proteins of the regulatory network; therefore, a synergistic approach is highly preferable that can switch off the action of harmful proteins, which in turn targets whole molecular networks that underlie the disease state [61,62].

Integrating network biology with polypharmacology can broaden current views on druggable targets while also aiding in understanding the pharmacological action of herbal medicines [13,63,64]. Polypharmacology and synergism discoveries are laying the groundwork for drug discovery in the following modern era of big data. Polypharmacology broadens the scope of drug discovery [65,66,67]. Molecules are linked to one another in orderly fashions that are characterized by strong binding affinities. Polypharmacology integrated with breakthroughs of structural biology and chemoinformatics has paved the way to develop licensable drugs with no side effects [68,69].

Network pharmacology could be an excellent place to start. The term “network pharmacology” mainly emphasizes the existence and importance of multi-targeted drugs instead of single-targeted drugs [70,71]. Hopkins recommended three approaches to develop a multi-target therapy: He began by prescribing a multidrug made up of many individual drugs. Designing of a multi-component drug was the second-most-important postulate. Designing a single drug acting on multiple targets was the last option. As per Hopkins’ view, the last postulate is advantageous since it would make dosage trials easier [13,63]. Virtual pharmacology and in silico analysis could be useful new tools in herbal medicine research. The underlying pharmacology, in particular, should be a primary consideration. More rigorous investigations on the bioavailability of natural products will undoubtedly lead to progress before these chemicals are used in in vitro experiments. Furthermore, research into the pharmacodynamics of natural compounds is critical in understanding pharmacological synergies and the potential network pharmacology of medicinal plants [12,72,73].

## 5. Methodology of Network Pharmacology Research

**Figure 2:**
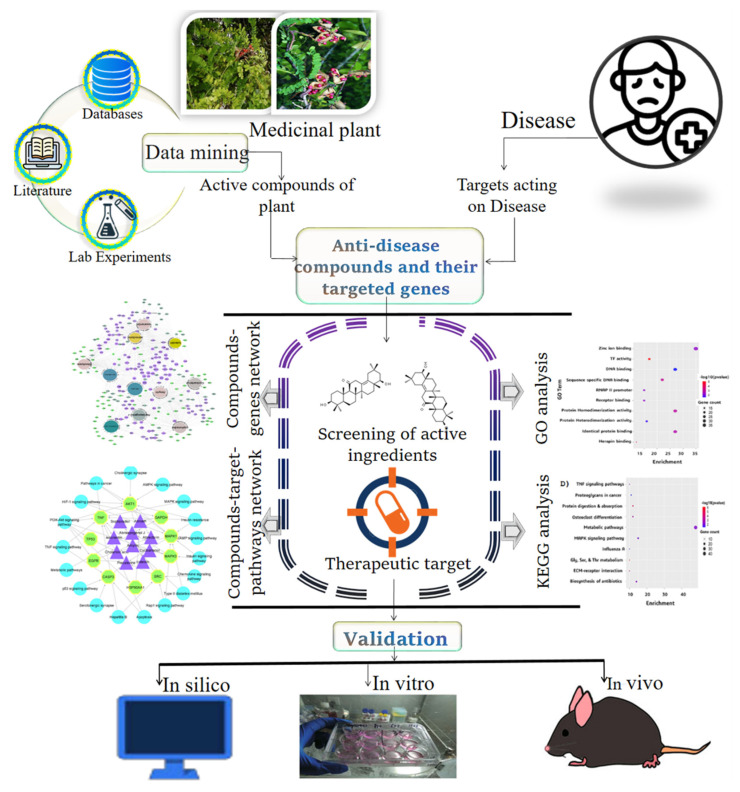
Graphical synopsis of network pharmacology research for the discovery of herbal medicines-derived targets, effect prediction, mechanism clarification, and new drug assistant discovery using network pharmacology approach. It analyzes the information from public data, high-throughput experimental data, and herbal medicinal data and constructs network using technologies of network expansion, optimization, comparison, knockout, and addition. Finally, it carries out computational and experimental verifications.

Network pharmacology research revolves around identifying compound- and disease-related genes, constructing a protein–protein interaction (PPI) network, and lastly, analyzing and visualizing the network [74,75,76]. The construction of molecular networks from large databases is a simple start. Then, using network analysis, key nodes are identified and key biological pathways are predicted [77]. Finally, additional network validation is performed to successfully validate the interaction between highly active constituents and their putative targets [78,79]. In this review, we highlighted all the steps of network pharmacology research. The workflow is displayed in Figure 2.

### 5.1. Data Mining

Identifying active compounds of medicinal plants and diseases-related targets is the preliminary step in network pharmacology research. Generally, a literature search is carried out to identify active compounds; however, various public databases provide a user-friendly interface to predict the active compounds of the medicinal plant [9,12]. After obtaining active compounds, the canonical SMILES of active compounds are retrieved from available public databases. Some online and standalone tools are available, which become a handy platform for identifying canonical SMILES [80]. After obtaining canonical SMILES, network pharmacology research turns around gene prediction or from canonical SMILES. To perform this task, a list of user-friendly tools and databa.ses are available. Another important thing is that we can get statistically significant genes by applying precise cutoff on the probability of genes and obtaining highly significant genes. The prediction of disease-related genes is a preliminary step to explore the molecular mechanism of medicinal herbs for treating multiple diseases and disorders [81,82]. Additionally, instead of relying on literature data, the target gene can also be obtained through real experiments; for example, researchers have now analyzed the transcriptome-wide gene expression microarray profiles of isolated cells in response to the exposure to plant extracts, their combinations, or purified compounds, followed by ingenuity pathway analyses in silico to elucidate their mechanisms of action and activated molecular networks behind predicted therapeutic effectiveness in various health conditions [83,84,85,86,87,88,89,90,91,92,93].

### 5.2. Network Construction and Analysis

Venn diagrams tools are preferable for identifying overlapped targets of diseases and compounds. This step mainly aims to predict disease-related genes and subsequently identify the common genes between diseases and compounds. The common genes are initial touchstones for further screening [94,95]. Network analysis is carried out to understand the mechanism of medicinal plants in disease treatment. Protein–protein interactions (PPI) are highly significant by virtue of having a high versatility, adaptability, and specificity [78,96]. The PPI network of key targets (common genes) is obtained through databases that provide the functional interactions among key targets [96,97]. Later, the network analysis is performed to predict the hub genes that have the best degree of connectivity. Biological networks supply us with a wealth of data [98]. The important point is how to retrieve key information from networks. Network analysis tries to uncover important targets, active ingredients, and their associated pathways by identifying targets. Network analysis employs a variety of methodologies, the most common of which is network functional analysis. Biological networks have been discovered to have a modular aspect, and many beneficial drugs have therapeutic effects by modulating several proteins instead of using a single protein. Several subnetworks having particular roles and topologies in large and complicated networks have been unveiled via topological research. At a functional level, GO enrichment analysis and KEGG pathway analysis provide exclusive key target features by exploring their associated pathways [43].

### 5.3. Validation of Results

It is important to verify the results obtained through the aforementioned steps. Various validation methods are available to confirm the efficacy of predicted molecular targets. In vitro and in vivo are generally considered the most viable methods, but these methods are time-consuming and require a high cost to yield correct results. However, with the emergence of high-throughput technologies and advancement in the genomic era, various in silico approaches have been designed, providing a handy platform for validation of results [99]. Lastly, both experimental and virtual methods are available to validate the predicted results.

Receptor–ligand molecular docking is used to predict the docking sites of active ingredients and key targets derived from network pharmacology. Therefore, network pharmacology and molecular docking effectively bridge the gap between western medicine and herbal medicine and greatly facilitates mechanistic studies on the synergistic actions of herbal medicines. Molecular docking has become a lightning rod and the most applicable approach in the drug discovery toolbox [100,101]. Molecular docking enables the prediction of interaction that ties up ligands with their corresponding proteins in a bound state [102]. Most researchers used the molecular docking approach for validation [96,103]. Mainly docking score and binding energy are considered key criteria for constituent screening. A list of studies has witnessed the importance of molecular docking as a validation technique in network pharmacology.

Zhang et al. [104] performed molecular docking to screen out the putative targets of *Prunella vulgaris* L., which can lower the risk of breast cancer. Docking analysis successfully predicted the strong binding affinity between active constituents of *Prunella vulgaris* L. and binding pockets of target proteins. In the work of Liu et al. [105], combined network pharmacology and molecular docking analysis were performed to uncover the molecular targets and mechanism of Huangqi Guizhi Wuwu decoction for treating rheumatoid arthritis. Through network analysis, a total of 790 compounds was obtained. Later molecular docking analysis revealed that out of 790 compounds, quercetin, kaempferol, and beta-sitosterol have a strong binding affinity with target proteins (VCAM1, JUN, and CTNNB1). Therefore, quercetin, beta-sitosterol, and kaempferol exhibited therapeutic effects on molecular targets and their associated pathways. Furthermore, Ruan et al. [106] uncovered the action mechanism of Dayuanyin for the treatment of COVID-19 by employing network pharmacology. Based on network analysis, they used molecular docking to validate predicted results. Molecular docking analysis revealed the binding affinity of active ingredients, namely naringenin, kaempferol, formononetin, quercetin, isofavone, and 7-Methoxy-2-methyl with core target genes (Interleukin 6, Interleukin 1B, and CCL2). In conclusion, molecular docking mainly aims to validate the successful activity of the active compounds against potential gene targets. The information obtained through molecular docking might aid researchers in understanding many aspects of biomedicine, from protein synthesis to health and disease.

Gene expression microarray analysis is also an un-doubtful technique for validating predicted results. Gene expression microarray analysis is the measurement of the activity of thousands of genes at once to provide a global view of cell processes. These profiles can be used to differentiate cells that are actively dividing or to illustrate how cells respond to a certain therapy. High-density microarrays are among the most powerful and versatile methods for analyzing the expression patterns of huge numbers of genes across different tissues or within the same tissue under various experimental circumstances. The widespread use of microarray technologies generates vast amounts of data, which encourages the development of improved analytical methods to anticipate the activities of target genes. To examine the differential gene expression levels of putative targets, gene expression data are downloaded from Gene Expression Omnibus (GEO) [107]. GEO is a freely available public repository of the National Center for Biotechnology Information (NCBI), which encloses the gene profiles. Only those genes are significantly expressed with a logFC value ±1 with an adjusted *p*-value < 0.05. If the logFC value is negative, then that gene is marked as downregulated, and a gene having a positive logFC value is called upregulated. Hence, these profiles are utilized at various phases of the network pharmacology process, which aid in identifying new drug targets, predicting novel gene activity, and understanding individual drug response variability.

Researchers use microarray analysis to validate predicted results in some network pharmacology studies. After successfully performing microarray analysis, they move toward real time-polymerase chain reaction (RT-PCR) to validate the differentially expressed target genes identified after microarray analysis. RT-PCR is now a well-established method for detecting and quantifying target genes in clinical diagnosis and treatment. One key application of this technology as a research tool is the rapid and accurate assessment of changes in gene expression due to pathophysiology, physiology, and development.

Hong et al. [108] predicted putative targets of flavonoids from citrus to treat non-alcoholic fatty liver disease by employing a network pharmacology approach. They used microarray analysis and RT-PCR for the validation of predicted results. They finally predicted VEGF-C as a key target of pure flavonoid from citrus to treat non-alcoholic fatty liver disease in mice. Zhang et al. [109] used a network pharmacology-based methodology to predict active constituents of Huangqi decoction against rat liver fibrosis. After performing network analysis, they used both gene expression profiling analysis and RT-PCR to validate the results. They finally demonstrated the strong actions of Huangqi decoction against rat liver fibrosis. Furthermore, Li et al. [110] also used the same methodology and revealed the targets of Sinomenine for the treatment of breast cancer.

Western blotting is another unquestionable and reliable technique for the validation of expression levels of target genes. Researchers commonly use Western blotting to validate the results derived using the target–pathway interaction network. The accuracy and reliability of results provided by Western blot analysis increase the confidence of researchers, which ultimately leads to make ground-breaking discoveries in the field of drug discovery and development. A list of studies evidenced the accuracy of Western blot analysis. Cai et al. [111] used Western blot analysis to validate results derived after network analysis. They finally concluded that Yinchenhao decoction suppresses the rat liver fibrosis involved in regulating multiple targets, especially affecting the apoptosis-related signalling pathways. Guo et al. [112] used Wu-Tou decoction to treat rheumatoid arthritis. For validation of results, they used Western blotting. Their study revealed that Wu-Tou decoction plays an important role in inhibiting inflammatory response in rheumatoid arthritis and is closely connected with the modulation effect of Wu-Tou decoction on the CCR5 signalling pathway in macrophages. Furthermore, wang et al. [113] investigated the multi-targets mechanism of triphala on cardio-cerebral vascular diseases by employing network pharmacology and Western blot analysis. Their study revealed that pharmacological mechanism and complicated components of Triphala, which could provide a theoretical basis for the research and development of new drugs for treating cardio-cerebral vascular diseases.

Generally experimental verifications such as in vivo analysis are mandatory in many studies in order to deeply analyze the results [114,115]. Based on the complicated nature of diseases and validation processes, researchers worldwide explored a new strategy to improve the efficiency of active ingredients screening, which ultimately helps in the discovery of some multi-target compounds with biological activity for the development of novel drugs against disease. Nowadays, researchers use the mouse model to successfully validate predicted results and make innovative treatment options against the deadliest diseases. In network pharmacology research, the effect of active compounds of medicinal plants on key disease signalling pathways are validated using a mouse model.

Qin et al. [12] used network pharmacology to predict the mechanisms of action of Shenkang in chronic kidney disease. They performed both pharmacological network analysis as well as in vivo validation for determining the potential effect and mechanisms of Shenkang in the treatment of chronic kidney disease. Their study proposed that Shenkang exerted a curative effect on chronic kidney disease and chronic kidney disease-related diseases by targeting different organs, proliferation processes, and regulating inflammation. Furthermore, Liu et al. [116] employed both network pharmacology and in vivo validation to understand the pharmacological mechanism of the Xianglian pill against ulcerative colitis. Their study revealed the clinical treatment efficacy of the Xianglian pill for ulcerative colitis.

Lastly, the quantification of validated data can be performed using several integrated approaches such as proteomics, transcriptomics, genomics, metabolomics, and high-throughput screening (HTS) [117]. HTS is an efficient approach to the modern era. HTS is a method for scientific experimentation that is particularly useful in drug development and is applicable to system biology, chemistry, and many other fields. HTS technology can swiftly discover billions of data samples and predict the effect of chemicals/compounds on specific molecular pathways [118,119]. Furthermore, this dual and novel high-throughput technology enables network data collection from experiments/trials and ultimately validates the network model’s accuracy. For instance Fakhari and Dittmer developed PCR chip technology to analyze gene expression [120]. The findings revealed that the method was suitable for high-throughput research. Another method is to validate the molecular interaction that exists between networks. This method yields a new perspective in the context of understanding drug activity mechanisms and the validation of the drug network or predicted model. It mostly consists of surface plasmon resonance (SPR) and biolayer interferometry (BLI) technologies, which can aid researchers in discovering the interaction between drugs and biomolecules [121,122]. High-throughput, high-precision, and real-time detection are all used in BLI and SPR techniques.

In short, network pharmacology along with validation techniques help in the discovery of key pharmacological mechanisms of herbs/herbal formulae. These integrated approaches lay a foundation for treating complex diseases by using medicinal plants. Therefore, more innovative and novel strategies must be applied to fully understand the therapeutic mechanisms of medicinal plants.

## 6. Research Approaches of Network Pharmacology

Network pharmacology can affect drug discovery and development in two ways: One method is to create a realistic network model and forecast the pharmacological target using public datasets or data from previous studies. Following that, the network equilibrium concept should be investigated through the mechanisms of functional drugs. Gu et al. [123] evaluated the impact of Rheidin A and C, along with Sennoside C, using this technique, and it was the first study on multiple integrated drugs for type 2 diabetes. The alternative strategy uses bioinformatics approaches and high-throughput technologies to construct a “drug–target–disease” network. The drug’s action in various biological processes was investigated by comparing the drug’s interaction with the network. In the literature, there are numerous examples of network pharmacology being used in drug development. For example, Li et al. [124] employed the Liuwei Dihuang tablet to forecast the best network targets and discovered that multi-layer networks could underpin the integrated action mechanism of herbs and herbal formulae. Furthermore, it has been shown that salvianolic acid B was appropriate and viable for treating cardiovascular disease by combining the previous study methodologies [125]. In conclusion, advances in systems biology and bioinformatics change our understanding of the treatment and diagnosis of diseases through medicinal plants from a network pharmacology perspective and ultimately contribute substantially to the modernity of medicinal plants.

## 7. From Network Pharmacology to Integrated Multi-Omics Approaches

Recent advancements in sequencing technologies have countered the revolution in various integrated approaches, in which the one named omics is the emergent field. With time, biology gradually depends on the data derived from multi-omics data [126,127,128]. Multi-omics data aimed to point out the interrelation among biomolecules, hence multi-omics data has paved the way for understanding function as well as interrelation among biomolecules [129,130]. Network pharmacology has evolved into a strong method for uncovering complicated biological interactions systematically. Network pharmacology uses “omics” techniques to detect variations at core molecular and cellular levels in terms of a specific pathophysiology or pharmacological treatment [9,131,132]. The obtained dataset aids in the generation of networks that describe molecular processes from the genetic to the metabolomics level. The main omics approaches used in network pharmacology are epigenomics, transcriptomics, proteomics, and metabolomics. Because there are so many molecular pathways and epigenetic effects on the phenotypic expressions of diseases, hence, a systems biology approach based on innovative modeling approaches is essential for investigating gene–environment interactions and therapy success [132,133,134]. The study of epigenetic alterations has yielded a wealth of information about prognostically important genes.

Several studies have been made on the mechanisms adopted by a cell to perform its functions better. As cells are comprised of the same set of genes, why does each gene behave differently from one another? Here comes a phenomenon named epigenetics. For a stage-by-stage characterization of epigenetic genes, a systems biology method was used, and epigenetic sub-network analysis identified a collection of conserved genes. Thus, the integration of network pharmacology demonstrated the existence of epigenetically de-regulated functional hotspots, which ultimately helps in fighting against disease by understanding the underlying mechanism in the progression and pathogenesis. For example, Gnad et al. [135] utilized differential gene expression as well as correlation network analyses to find dysregulated epigenetic regulator genes in cancer and found EZH2 to be the most significantly overexpressed epigenetic regulator in cancer that was classified as an oncogene. Furthermore, the bioinformatics analysis of single nucleotide polymorphisms (SNPs) may aid in the prevention of adverse drug reactions in certain patient groups and develop new medical interventions.

## 8. Merging the Molecular Disease Network with the Pharmacological Network of the Candidate Drugs

The biological clock of life has recently been progressively investigated from a systems approach across fields of science and technology. There has already been a lot of work conducted to develop practical frameworks for bringing “systems thinking” to improve public health. The minimum distance among proteins was evaluated in the published paper, which highlighted the links between drug targets and disease-target products. Considerable differences were discovered between analgesic and etiological medicines, and the research revealed a modern trend towards rational drug design. The integration of disease-related targets and drug targets through network biology has become a crucial step in developing novel and putative drugs [136,137,138].

In contrast, drug repurposing, which uses old drugs to develop new and better drugs, would be possible with network pharmacology methodologies. A new pharmaceutical product takes at least ten to fifteen years to develop and costs between $500 million and $2 billion, but there has been a steady rate of introduction of new drugs. Existing drugs already have clinical evidence, so getting them approved for a new use takes very little energy and money [139,140,141]. Alternatively, we may use other techniques to screen out existing drugs and analyze which herbal formula or herb has the potential to act as a drug. As a result, we offer an approach for the repurposing of old drugs based on network pharmacology.

## 9. Implications of Network Pharmacology for Therapy

The widespread failure of specific candidate drugs to progress from pre-clinical to clinical trials raises the question of whether a particular drug discovery is the best technique. Due to the poor understanding and validation of these targets in patients, even using medications operating on defined targets coupled to robust biological networks is difficult [142]. As a result, network pharmacology is becoming increasingly essential, and it is attracting a lot of interest in contemporary drug discovery [3]. For example, pleiotropic active ingredients targeting numerous proteins and biological processes in cancer-related networks could be effective. Herbal medicinal treatments are used all throughout the world to keep people healthy. Due to their diversity in structure, bioactivity, and nontoxicity, herbal medicines are recognized as a significant fountainhead for new active molecules in drug discovery, drawing global attention [143]. The paradigm of “one disease, one drug, one target” is giving way to “one disease, one drug, several targets”. Network pharmacology examines when and where one target can suppress disease characteristics, such as tumor progression, leading to the development of medicines that do not induce side effects. Network pharmacology’s benefits have become incredibly influential in drug discovery, particularly in repurposing old drugs [144]. Computational drug designing aided by network-based methodologies helps in predicting the adverse effects of drugs and helps the drug molecule find its target binding site [145]. Network pharmacology offers new drug discovery options that may be more fruitful than using herbal medicine without any scientific basis. This hypothesis drew interest since it offered the possibility of having efficient therapies that were more targeted and had less adverse effects in normal body cells. Network pharmacology may offer novel options for rigorous target selection and the development of multi-target, distinctive active compounds to treat them [28]. In the PPI network, highly linked regions are highly preferable because these nodes are considered the main target in the disease state. Hence, by targeting such nodes, we can achieve the goal. On the other hand, drugs cannot block all targets in a regulatory network. Only around fifteen percent of nodes in a particular network are druggable [146,147]. Multiple approaches to generating appropriate phyto-therapies based on network data can be considered:

- If the potentially active compounds of herbs or herbal mixes are identified, they can be considered. This technique is mostly made based on their use in herbal medicine. Herbal formulations are similar to multi-drug targeted therapy [51].
- Active compounds can simply be used to achieve multi-target specific therapy using selective poly-pharmacological methods [148,149]
- Proteins that are not required in normal cells could become therapeutically important if they’re linked together in a cancer network. Their simultaneous eradication or inhibition could result in more effective or even synergistic tumor cell eradication. What makes perfect sense in the human physiological process is to create significant therapies options. A potential answer to this difficulty could be to use polypharmacology to disrupt whole disease-causing networks using botanicals or sophisticated herbal mixes that target numerous targets, rather than knocking out specific proteins [150,151].

## 10. Databases and Data Analysis Tools Related to Network Pharmacology

Biologically important databases that provide a huge amount of data related to the relationship between biomolecules enable the researcher to use network pharmacology as a modern era drug discovery approach (Table 1). All these databases and tools are freely available, and providing a free hand to users can be useful in retrieving valuable information from the perspective of network pharmacology research.

## 11. Application of Network Pharmacology: From Understanding of Complex Interactomes to the Design of Multi-Target Specific Therapeutics from Nature 

Despite the research and development in pharmaceutical industry, the dramatic drop in the number of new treatments options raises the question of whether single-targeted drug discovery is a felicitous approach or not. In such scenarios, network pharmacology approaches are extremely valuable, as they differ from traditional drug discovery approaches by addressing the potential of drugs to target several proteins or networks involved in a disease [197]. Furthermore, employing high-throughput screening and bioinformatics aids in the construction of predicted drug–target disease network models. Such approaches help to explore the underlying mechanisms of drug actions on biological networks by comparing the interaction of a drug with its respective target model. The knowledge of multi-facetted pathway interactions considerably strengthened with recent advancements in network biology. Therefore, network pharmacology is solidifying its position in the treatment of the deadliest diseases and disorders.

**Figure 3:**
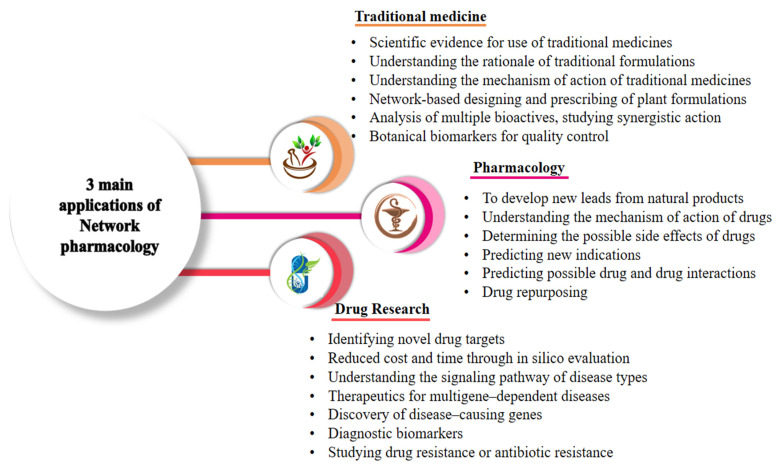
Three main application of network pharmacology in health biology for exploring the basic pharmacological effects of drugs on diseases and their mechanisms.

Network pharmacology discerns the protein–protein interactions associated with clinical outcomes of particular diseases and disorders. Nowadays, researchers are merging multi-omics approaches with computer technology to precisely record the unified metabolic response in humans to study an increasing number of complicated disorders. Below, we discuss some exclusive applications of network pharmacology in biomedical sciences. Figure 3 presents important applications of network pharmacology.

### 11.1. A Pneumonia Outbreak Associated with a New Coronavirus of Probable Bat Origin

SARS-CoV-2 is a β-coronavirus belonging to order Nidovirales and family *Coronaviridae*. The other β-coronaviruses, including Severe Acute Respiratory Syndrome [198] in 2002–2003 and Middle East Respiratory Syndrome [199] in 2012–2013, have also been reported in the past two decades [200,201,202]. However, COVID-19 is a large-scale pandemic of the 21st century and an alarming public health issue. Numerous clinical research endeavors have revealed that traditional medicines have a substantial effect on COVID-19 treatment, offering new promise for the management of COVID-19. Using network pharmacology, herbs/herbal formulae could also be incorporated in the COVID-19 diagnosis and treatment protocols. Numerous studies have been conducted that used network pharmacology to screen out the active compounds of medicinal plants for the treatment of COVID-19. Jin et al. [203] used Xuebijing injection, Wang et al. [204] used Qingfei Paidu decoction, and Li et al. [205] used Lianhua Qingwen and found that these formulae and medicines are viable to be used against COVID-19 treatment. As a result, it served as a starting point for a more in-depth exploration of the cornerstone of antiviral granules and a novel treatment concept for COVID-19.

Tao et al. [100] employed network pharmacology and molecular docking to understand the action mechanism of the Huashi Baidu formula against COVID-19. Their findings proposed that active compounds of the Huashi Baidu formula control numerous signalling pathways via ACE2, which might play a therapeutic role in treating COVID-19. These pathways include the MAPK signalling pathway, TNF signalling pathway, and the PI3K–Akt signalling pathway. Further molecular docking revealed that baicalein and quercetin were the top two compounds, indicating that both compounds could play a significant role in treating COVID-19. Another research performed by Zhang et al. [206] demonstrates the chemical compounds present in the lung-cleaning and toxicity-excluding (LCTE) soup for the treatment of COVID-19 in a network pharmacology perspective. Their findings suggest that the LCTE soup contains ingredients that have the ability to directly inhibit the progression of COVID-19. Moreover, LCTE targets the pathways that are mainly involved in viral and other microbial infections, inflammation/cytokine response, and lung diseases. Their research provides a biological foundation for employing LCTE soup to treat COVID-19 and its symptoms. Furthermore, Niu et al. [74] studied the action mechanism of three medicines (Jinhua-Qinggan Granule, Xuebijing Injection, Lianhua-Qingwen Capsule) and three herbal formulae (9 *HuaShi-BaiDu Formula, Qingfei-Paidu Decoction, and XuanFei-BaiDu Granule) against COVID-19 by using a network pharmacology approach. Their findings suggested that these three medicines and three formulae has been shown to have a positive effect on the prevention and rehabilitation of COVID-19 in at-risk individuals. They proposed that luteolin, ursolic acid, quercetin, and rutin could inhibit the progression of COVID-19 by downregulating the interleukin-6. Finally, we conclude that medicinal plants contain pythochemicals that have the ability to directly suppress the COVID-19, target proteins associated with common COVID-19 symptoms and influence the disease-related major pathological processes.

### 11.2. Cancer

Cancer is indeed one of the causes of morbidity and mortality in humans and remains a significant major health concern worldwide. Cancer can be efficiently addressed as a multifactorial disease by modulating various targets and carcinogenic signalling pathways [207,208]. As in the case with cancer, single-targeted drug discovery has shown to be unsuccessful in combating complicated systemic diseases with complex biological systems [142]. In these scenarios, network pharmacology approaches are extremely valuable since they vary from traditional drug discovery by addressing multi-targeted drugs that ultimately target many proteins or networks involved in the progression or development of disease states [131]. Techniques based on network pharmacology have the potential to dramatically minimize the difficulties in routine clinical research, such as patient heterogeneity in population and disease. Furthermore, cancer is heterogeneous, primarily characterized by the existence or lack of relevant therapeutic targets. There are currently very few treatment approaches available for heterogeneous populations with heterogeneous malignancies [209,210]. As a result, there is a pressing need to create network pharmacology-based methodologies to change the current concept of drug designing and increase our understanding of the mechanism of action. The proper application of network pharmacology-based methodologies may help to avoid problems in cancer drug therapy and speed up the discovery of new anticancer medicines. Moreover, network-based studies proposed target genes as promising and viable therapeutic targets to reduce the *incidence* of cancer, thereby exerting potential therapeutic effects in cancer.

A HER2-positive breast cancer is one that tests positive for the protein human epidermal growth factor receptor 2 (HER2). This protein stimulates cancer cell proliferation. Cancer cells in around one out of every five breast tumors have extra copies of the gene that produces the HER2 protein. Zeng et al. [211] used a network pharmacology-based methodology to explore the pharmacological mechanism of Yanghe decoction against HER2-positive breast cancer. They proposed quercetin, luteolin, and naringenin as key active ingredients of Yanghe decoction that may have anticancer properties. Their findings successfully predicted, illuminated, and confirmed the molecular synergy of Yanghe decoction for HER2-positive breast cancer.

Zhen et al. [212] explored the active ingredients as well as important pathways of Shen-qi-Yi-zhu decoction against gastric cancer. In a nutshell, their research demonstrated that a combo of network pharmacology and in vitro studies clarifies the efficient and beneficial molecular mechanism of Shen-qi-Yi-zhu decoction. They also proposed that Shen-qi-Yi-zhu decoction plays an anti-tumor role by inhibiting the PI3K/AKT/mTOR pathway. The PI3K/AKT/mTOR pathway is found in almost all tumors and plays a key function in cancer biology. Hence, the herb/herbal formulae played an important role in the anti-tumor area.

Colorectal cancer, a silent monster, is indeed the leading cause of cancer-related death. Liu et al. [213] used a network pharmacology approach for the identification of action mechanisms of *Hedyotis diffusa* against colorectal cancer. Network analysis revealed that *Hedyotis diffusa* exhibited a promising therapeutic impact on colorectal cancer by targeting tumor-associated signalling pathways. This provides a foundation for understanding the anti-colorectal cancer activity of *Hedyotis diffusa.* Song et al. [214] used the same plant (*Hedyotis diffusa)* for uncovering the multi-target pharmacological mechanism on prostate cancer. Therefore, it has become clear that using network pharmacology, we can screen the active compounds of single herb/herb formulae for the treatment of more than one disease. Bing et al. [215] used bioinformatics and network pharmacology approaches to investigate the mechanism of Fuzheng Kangai for lung cancer treatment. Furthermore, Meng et al. [216] incorporated a network pharmacology approach with molecular docking to uncover the molecular mechanisms of Kushen injection to treat lung cancer. In short, network pharmacology yields a new perspective in understanding the action mechanisms of herb/herbal formulae for the treatment of various types of cancer.

### 11.3. Cardio-Cerebrovascular Diseases (CCVDs)

Cardiovascular and cerebral vascular diseases have become some of the world’s most serious health problems [217]. Botanical drugs, of long-used medicinal plants, have been shown to provide many benefits for CCVD treatment [205,218]. However, the molecular mechanisms underpinning medicinal plants’ ability to heal CCVD are still unknown. Currently, a novel systems-pharmacology platform named network pharmacology has been proposed to comprehensively understand the pharmacological mechanism of medicinal plants for the treatment of CCVDs by merging pharmacokinetic screening, target identification, and network analysis. This approach offers a new paradigm for systematically understanding the mechanism of herb/herbal formulae against CCVDs.

In the light of network pharmacology, Yang et al. [219] elaborated on the active compounds of *Ginkgo biloba* leaves, their potential target, and associated pathways for treating CCVD, hence providing a theoretical basis for additional experimental research. Their findings revealed that *Ginkgo biloba* leaves exhibit a protective effect on CCVDs, most likely by regulating various processes and attacking multiple targets linked to a variety of biological pathways. Their study provides an important reference for understanding the efficacy of *Ginkgo biloba* leaves in the treatment of CCVDs and a fresh technique for discovering new medicines from plants. Ren et al. [220] used herbal formulae for the treatment of stroke. In their work, screening results represented various bioactive compounds that played a decisive role in treating stroke by targeting the disease-related genes.

Tao et al. [221] employed a network pharmacology-based methodology to predict active ingredients along with putative targets of the *Radix Curcumae* formula for the treatment of CCVDs. Their study systematically demonstrates the mechanism of the *Radix Curcumae* formula in the treatment of CCVD, while also predicting potential targets to facilitate the development of candidate herbal drugs in future work. Wang et al. [222] explored the active compounds of *Salvia miltiorrhiza Burge.* and *Carthamus tinctorius* L. for the treatment of CCVDs. Their study revealed that *Salvia miltiorrhiza Burge.* and *Carthamus tinctorius* L. may promote cerebral blood flow by dilating blood vessels, reducing neurotoxic damage, and protecting brain tissue from free radical damage.

Cui et al. [223] employed a network pharmacology approach to uncover the mechanism of Shuxuening injection against ischemic stroke. Their findings demonstrated that by suppressing inflammation and regulating the degree of oxidative stress, Shuxuening injection could treat ischemic stroke and minimize the death of neuron cells in brain tissue, thus safeguarding the brain tissue of rats. Their study combined network pharmacology, molecular docking, and animal experiments to provide the first coherent and detailed investigation of Shuxuening injection mechanism for the treatment of ischemic stroke and comprehends the multi-component and multi-target synergy mechanism of Shuxuening injection [223].

Furthermore, Chen et al. [224] identified the active compounds and putative targets of the Yangxinshi tablet to treat heart failures by using network pharmacology research. Their analysis revealed the cardiovascular protective effect of the Yangxinshi tablet, which was primarily enriched in immune and cardiovascular systems. The network-based study could aid researchers in simplifying the complex mechanism of the Yangxinshi tablet. It may also provide a realistic method for determining the chemical and pharmacological foundations of other herbal formulae.

### 11.4. Diabetes Mellitus

Diabetes mellitus (DM) is a pandemic of the 21st century and is a rapidly growing global problem [225]. DM is associated with the diverse interplay of genetic, environmental, and behavioral risk factors [226]. People with DM are more susceptible to a variety of short- and long-term problems, leading to serious complications [227]. Network pharmacology consisting of natural products is seen as a viable therapeutic method for DM and could provide answers to the questions raised above. Wang et al. [228] employed a network pharmacology-based approach to explore the active ingredients of Astragaloside IV as a best treatment option against type 2 diabetes mellitus (T2DM). Recent findings demonstrated that docking analysis as well as network analysis might drastically cut preliminary screening expenditures and offer a complete analysis of the action mechanism in the development and discovery of novel drugs. Gu et al. [123] used a combination of network pharmacology and molecular docking studies to demonstrate the action mechanism of Tangminling tablets for the treatment of T2DM. The compound–compound network and compound target network demonstrated that over 100 chemical ingredients out of 667 in the formula could target 37 T2DM-related target proteins. The important ingredients in Tangminling tablets were anticipated, and a few of them have previously been described in the literature. Furthermore, due to their pharmacological actions, numerous chemical compounds, particularly procyanidin C1, Rheidin A, Rheidin C, Sennoside C, and Dihydrobaicalin, were important and used as anti-diabetic candidates.

Sorghum bicolor is rich in anti-diabetic bioactive constituents and is a plausible resource of anti-diabetic ingredients. Oh et al. [229] employed network pharmacology to identify active compounds of sorghum bicolor having the potential to treat diabetes mellitus. Their findings imply that essential active ingredients are present in sorghum bicolor, which may help to reduce the severity of T2DM by activating the peroxisome proliferator-activated receptors’ (PPARs) signalling pathways. According to the results of their study, the anti-diabetic activity of sorghum bicolor can be linked to four main compounds (alpha-sitosterol, propyleneglycol monoleate, campesterol, and 25-Oxo-27-norcholesterol) that are highly associated to the PPAR signalling pathway.

In the work of Zhou et al. [230], a network pharmacology-based methodology is used to analyze the mechanism of Xiao Ke Yin Shui for the treatment of T2DM. Their study proposed that proteins such as protein kinase B, phosphatidylinositol 3-kinase, and tumor necrosis factor are primarily regulated by compounds present in Xiao Ke Yin Shui’s formula. Therefore, the Xiao Ke Yin Shui formula has synergistic therapeutic benefits and has an anti-diabetic impact primarily via lowering insulin resistance.

### 11.5. Neurodegenerative Diseases

Neurodegenerative diseases are conventionally demarcated as progressive degeneration and/or death of nerve cells. Neurodegenerative diseases have a great diversity of clinical symptoms that vary widely in disease status and prognosis. However, due to insufficient diagnostic methods, the patients are diagnosed, on average, at the middle or late disease stage, leading to a poor prognosis. The identification of potential biomarkers that can stop disease pathogenesis and serve a virtual shortcuts will be hailed as the sensation of the current era.

Medicinal treatments consisting of natural products are considered promising and fabulous treatment options for neurodegenerative diseases. By virtue of their component diversity, higher multi-target activity, and lower toxicity, herbs are becoming a dominant contributor for developing multi-target drugs. Recently, Zhou et al. [231] used a network pharmacology-based strategy to investigate pharmacological mechanisms of *Tinospora sinensis* for the treatment of Alzheimer’s disease. Their findings demonstrate that *T. sinensis* had a significant effect on the expression of protein PI3K and Akt; hence, *T. sinensis* could prevent and treat Alzheimer’s disease through a multi-compound–multi-target–multi-pathway regulatory network.

Parkinson’s disease is another major neurodegenerative disorder, following Alzheimer’s disease, that imposes a serious burden on families and even the whole society. Li et al. [232] applied a network pharmacology-based approach to study of the molecular mechanisms of Shaoyao Gancao decoction in treating Parkinson’s disease. Their study proposed 48 bioactive constituents mediating 30 Parkinson’s disease-related targets to exert synergism, and the same target can be enriched in multiple signal pathways and biological processes, implying that the decoction can exert a synergistic effect on Parkinson’s disease via multi-targets and pathways. These findings shed light on the molecular mechanisms underpinning the efficacy of Shaoyao Gancao decoction in the treatment of Parkinson’s disease, therefore allowing researchers to dig further into herbal medicine for developing innovative and exciting therapeutic options against Parkinson’s disease.

Huntington’s disease is an autosomal-dominant, neurodegenerative disorder with a primary etiology of corticostriatal pathology. Currently, there are no treatments that can slow or stop the progression of the disease. Dai et al. [233] also employed the same network pharmacology-based methodology to explore a novel herbal formula against Huntington’s disease, which was then further validated by a support vector machine model. The authors demonstrated that *Brucea javanica*, *Dichroa febrifuga*, *E. micranthum Harms*, *Erythrophleum guineense*, *Holarrhena antidysenterica*, and *Japanese Ardisia Herb* contained active compounds that might be a novel medicine formula for Huntington’s disease.

Not only these, recently, Liu et al. [234] used a network pharmacology study on the triterpene saponins from *Medicago sativa* for the treatment of all types of eurodegenerative diseases. The findings of this study will serve as references (for active compounds, major protein targets, and signalling pathways) that can be used for the treatment of neurodegenerative diseases and future research. In the future, more studies are needed to explore the multi-target pharmacological mechanism of herbs against neurodegenerative diseases

Thus, understanding the herb/herbal formula using network pharmacology is an emerging trend of this era. The multi-target action mechanism of network pharmacology is compatible with the complicated nature of disease and drug action. Additionally, protein–protein interactions networks or interactomes have been commonly used to understand complex disease mechanisms. The network pharmacology approach, however, oversimplifies disease mechanisms, which are in fact complex subnetworks within the interactome. Beyond all the applications of network pharmacology mentioned above, a list of studies has been conducted on other diseases and is summarized in Table 2.

## 12. Limitation and Solution

Network pharmacology has proved to be beneficial in drug development, which aids in revitalizing herbal medicines. Although there are a few drawbacks of applying network pharmacology research in herbal medication, hopefully, they will be fixed in the future. For the identification of active ingredients and disease-related targets, network pharmacology depends on various public databases. Despite their curation, databases may have inconsistencies due to a variety of information sources, theories, and experimental results. Furthermore, herbs that encounter specific pre-trial procedures throughout their development have undergone various chemical changes—using contemporary, high-throughput techniques such as liquid chromatography is one solution to overcome this challenge.

ADMET profiling is used to validate the pharmacokinetic properties of the highly active constituent. ADMET analysis is a challenging process in drug discovery. Various in silico tools are available for ADMET profiling; however, experimental validation is necessary to validate active compounds’ pharmacokinetic properties.

The identification of putative targets depends on the one or usually one single database, owing to the limited availability of databases with unrestricted accessibility. This might sometimes lead to unsatisfactory consequences. Therefore, the integration of multiple databases is one solution to solve the challenge.

## 13. Conclusions

Medicinal plants represent a novel alternative and preferred treatment to handle ailments with no satisfactory remedy. For a long time, humans use herbal medicines to manage different diseases. The negative effects of synthetic medicines have demanded progress in the use of natural products for the management of disease. To achieve stunning gains in the future, the use of emerging technologies must be tied with a research basis. Most of the commercially produced medicines are derived from plants. This review of the current literature provides a comprehensive overview of drug discovery from medicinal plants by employing a network pharmacology approach. This review article is a touchstone for the initial screening of medicinal plants for treatments of various ailments, and additional spectra of phytochemicals provide baseline data about phytochemical studies. The network pharmacology approach establishes the latest scientific foundation for determining the efficacy of multi-component, multi-target compound formulae and exploring more therapeutic targets’ disease treatment. In summary, advancements in systems biology and bioinformatics will make an operational shift from reductionism in favor of network pharmacology. They will undoubtedly bring about a conceptual move in drug discovery and contribute to the modernization and globalization of herbal medicines. Different dynamic networks and quantitative networks may be another tendency, and more and more employment of network pharmacology technology will make the expenditure much less in the future. This review lays the groundwork for further research on the protective mechanisms of medicinal plants in disease treatments and the applications of network pharmacology in drug discovery.

## Figures and Tables

**Table 1:** List of available resources for network pharmacology research.

Sr. No#	Resources	Brief Description	Usage	URL	Reference
1.	BioCarta	Online maps of metabolic and signalling pathways	Database of gene interaction models	https://maayanlab.cloud/Harmonizome/dataset/Biocarta+Pathways (accessed 29 April 2022)	[152]
2.	BioGRID	Biological General Repository For Interaction Datasets	Retrieval of protein–protein interaction network	http://thebiogrid.org/ (accessed 29 April 2022)	[153]
3.	C2Maps	Computational Connectivity Maps	Annotation of drug–protein pairs	http://bio.informatics.iupui.edu/ (accessed 29 April 2022)	[154]
4.	CB	Chemical book	Retrieval of chemical structures	http://www.chemicalbook.com/ (accessed 29 April 2022)	[155]
5.	ChEMBL	Database of bioactive compounds	Retrieval of functional as well as binding information of active compounds	https://www.ebi.ac.uk/chembl/ (accessed 29 April 2022)	[156]
6.	ChemProt	Chemical–protein–disease annotation database	Analysis of interaction between chemical and protein	http://www.cbs.dtu.dk/services/ChemProt-2.0/ (accessed 29 April 2022)	[157]
7.	ChemSpider	Database of chemical structures	Retrieval of chemical structures	http://www.chemspider.com/ (accessed 29 April 2022)	[158]
8.	CHMIS-C	Comprehensive Herbal Medicine Information System for Cancer	Database of herbal medicine related cancer	http://sw16.im.med.umich.edu/chmis-c/ (accessed 29 April 2022)	[159]
9.	COGs	Clusters of Orthologous Gene	Classification of proteins on phylogenetic basis	https://www.ncbi.nlm.nih.gov/COG/ (accessed 29 April 2022)	[160]
10.	CPDB	Consensus Path DataBase	Molecular functional interaction database	http://cpdb.molgen.mpg.de/ (accessed 29 April 2022)	[161]
11.	Cytoscape	Database for network construction and visualization	Network analysis	https://cytoscape.org/ (accessed 29 April 2022)	[162]
12.	DAVID	Database for Annotation, Visualization & Integrated Discovery	Functional annotation	https://david.ncifcrf.gov/ (accessed 29 April 2022)	[163]
13.	DIP	Database of Interacting proteins	Analysis of protein–protein interaction network	http://dip.doe-mbi.ucla.edu (accessed 29 April 2022)	[164]
14.	DrugBank	Online database containing information on drugs	Analysis of detailed drug data	http://www.drugbank.ca/ (accessed 29 April 2022)	[165]
15.	GeneCards	Database of human genes	For identification of disease-related genes	https://www.genecards.org/ (accessed 29 April 2022)	[166]
16.	Guess	Computer program for the analysis and visualization of networks	Network analysis	http://www.levmuchnik.net/Content/Networks/ComplexNetworksPackage.html (accessed 29 April 2022)	[167]
17.	HAPPI	Human Annotated & Predicted Protein	Retrieval of protein–protein interaction network	http://bio.informatics.iupui.edu/HAPPI/ (accessed 29 April 2022)	[168]
18.	HIT	A comprehensive and fully curated database for linking herbal active ingredients to targets	Herbal ingredients’ targets identification	http://lifecenter.sgst.cn/hit/ (accessed 29 April 2022)	[169]
19.	HPRD	Human Protein Reference Database	Retrieval of protein–protein interaction network	http://www.hprd.org/ (accessed 29 April 2022)	[170]
20.	InterPro	Integrative database of protein families	Collection of protein families	http://www.ebi.ac.uk/interpro/ (accessed 29 April 2022)	[171]
21.	KEGG	Kyoto Encyclopedia of Genes and Genomes	Pathway analysis	http://www.genome.jp/kegg/ (accessed 29 April 2022)	[172]
22.	LookChem	Database of chemical structures	Retrieval of chemical structures	http://www.lookchem.com/ (accessed 29 April 2022)	[173]
23.	MetaCore^TM^	MetaCore (TM)	Pathway analysis	http://www.genego.com (accessed 29 April 2022)	[174]
24.	MMsINC	Database of chemoinformatics	Retrieval of chemical structures	http://mms.dsfarm.unipd.it/MMsINC/search/ (accessed 29 April 2022)	[175]
25.	NetMiner	Computer program for the analysis and visualization of networks	Network analysis	http://graphexploration.cond.org/ (accessed 29 April 2022)	[176]
26.	NetPath	Network pathway analysis	Pathway analysis	http://www.netpath.org/ (accessed 29 April 2022)	[173]
27.	NetworkX	Computer program for the analysis and visualization of networks	Network analysis	http://www.analytictech.com/ucinet/ (accessed 29 April 2022)	[177]
28.	OPHID	Online predicted human interaction database	Retrieval of protein–protein interaction network	http://ophid.utoronto.ca (accessed 29 April 2022)	[178]
29.	Pajek	Computer program for the analysis and visualization of network	Network analysis	http://pajek.imfm.si/doku.php (accessed 29 April 2022)	[179]
30.	PDB	Protein Data bank	Retrieval of protein related information	http://www.rcsb.org/pdb/ (accessed 29 April 2022)	[180]
31.	PDTD	Protein Database for Drug Target	Identification of drug target	http://www.dddc.ac.cn/pdtd/ (accessed 29 April 2022)	[181]
32.	PharmGBK	Pharmacogenomics knowledge base	Analyze the genes response to drugs	http://www.pharmgkb.org/ (accessed 29 April 2022)	[182]
33.	PubChem	Public repository for information on chemical substances	Analysis of chemical compounds	https://pubchem.ncbi.nlm.nih.gov/ (accessed 29 April 2022)	[183]
34.	PubMed	Public/Publisher MEDLINE	Literature review	https://pubchem.ncbi.nlm.nih.gov/ (accessed 29 April 2022)	[184]
35.	Reactome	Database of pathways, reactions, and biological processes	Pathway analysis	http://www.reactome.org (accessed 29 April 2022)	[185]
36.	SignaLink	Signalling pathway analysis resource	Pathway analysis	http://signalink.org/ (accessed 29 April 2022)	[186]
37.	SIRC-TCM	Shanghai Innovative Research Center of Traditional Chinese Medicine	Detailed analysis of traditional chinese medicine	http://www.tcm120.com/1w2k/tcm_species.asp (accessed 29 April 2022)	[187]
38.	STITCH	Search Tool for Interactions of Chemicals	Analysis of target–drug relationship and biological pathways	http://stitch.embl.de/ (accessed 29 April 2022)	[188]
39.	STRING	Search Tool for the Retrieval of Interacting Genes/Proteins	Retrieval of protein–protein interaction network	http://string-db.org/ (accessed 29 April 2022)	[189]
	SwissTargetPrediction	Estimate the macromolecular targets of a small molecule	Identification of compound related genes	http://www.swisstargetprediction.ch/ (accessed 29 April 2022)	[190]
40.	TCMGeneDIT	Database of traditional Chinese medicine, gene, and disease information using text mining	Detailed analysis of traditional chinese medicine	http://tcm.lifescience.ntu.edu.tw/ (accessed 29 April 2022)	[191]
41.	TCMID	Traditional Chinese medicine integrated database	Detailed analysis of traditional chinese medicine	http://www.megabionet.org/tcmid/ (accessed 29 April 2022)	[192]
42.	TcmSP	Traditional Chinese medicine systems pharmacology database	Detailed analysis of traditional chinese medicine	http://tcmspnw.com (accessed 29 April 2022)	[193]
43.	TD@T	Database of traditional Chinese medicine @ Taiwan	Retrieval of traditional chinese medicine related information	http://tcm.cmu.edu.tw/ (accessed 29 April 2022)	[173]
44.	TTD	Therapeutic Target database	Drug target identification	http://bidd.nus.edu.sg/group/cjttd/ (accessed 29 April 2022)	[194]
45.	Ucinet	Computer program for the analysis and visualization of networks	Network analysis	http://www.netminer.com/ (accessed 29 April 2022)	[195]
46.	UniProtKB	Universal protein knowledge database	Analysis of protein	http://www.uniprot.org/uniprot/ (accessed 29 April 2022)	[196]

**Table 2:** Application of network pharmacology for treatment of diseases using herb/herbal formulae.

Diseases	Herb/Herbal Formula	Reference
Asthma	Qingfei Xiaoyan Wan	[235]
	Zhike Chuanbei Pipa Dropping Pill	[236]
Breast cancer	Bushen Zhuanggu formula	[237]
	Yanghe decoction	[211]
Bronchial Asthma	*Schisandra chinensis*	[238]
	Ma Huang Tang	[239]
	Si Jun Zi Tang	[240]
Cardiovascular and cerebral vascular diseases	Nao Xin Tong	[241]
	Tong Xin Luo	[242]
	Dan Hong injection	[243]
	*Astragali radix*	[244]
	Liu Wei Di Huang pill	[245]
	Bai Hu Jia Ren Shen decoction	[246]
	Bu Yang Huan Wu decoction	[247]
Cardiovascular disease	*Panax notoginseng*	[248]
	*Salvia miltiorrhiza*	[249]
	Naoxintong	[250]
	Fufang Danshen formula	[251]
	*Ginkgo biloba* leaves	[219]
	*Radix Curcumae*	[221]
	*Salvia miltiorrhiza Burge.* and *Carthamus tinctorius*	[222]
	Shuxuening injection	[223]
Chronic bronchitis	*Eriobotrya japonica*	[252]
	Zhi Chuan Ling	[253]
Chronic obstructive pulmonary lung disease	Bu Fei Jian Pi Formula	[254]
	Yin Huang Qing Fei	[53]
	Tanshinone	[255]
Colorectal cancer	Hedyotis diffusa	[213]
COVID-19	Xuebijing injection	[203]
	Qingfeipaidu decoction	[204]
	Lianhuaqingwen	[205]
	Huashi Baidu formula	[100]
	Jinhua Qinggan Granule, Lianhua Qingwen Capsule, Xuebijing Injection, Qingfei Paidu Decoction, HuaShi BaiDu Formula, and XuanFei BaiDu Granule	[74]
Diabetes mellitus	Bu-Fei-Yi-Shen formula	[256]
	Xiao Ke Yin Shui	[230]
	*Erigeron breviscapus*	[257]
	Astragaloside IV	[228]
	Tangminling tablets	[123]
	*Sorghum bicolor*	[229]
	Xiao Ke Yin Shui	[230]
Dysmenorrhea of gynecology	Si Wu Tang	[248]
Fever and chill	Da Chaihu Decoction and Xiao Chaihu Decoction	[258]
Gastritis	Atractylodes macrocephala Koidz	[259]
	Arctigenin	[260]
Gastric cancer	Shen-qi-Yi-zhu decoction	[212]
Gout	Modified Simiao wan	[95]
Hepatocellular carcinoma, intestinal tuberculosis, and gastrointestinal inflammation	Gansui Banxia tang	[261]
Hepatocyte injury	*Fructus Schisandrae chinensis*	[262]
Hyperlipidemia	Cynarin	[263]
	Poncimarin, Hexahydrocurcumin, and Forsythoside C	[264]
Inflammation	*Folium eriobotryae*	[265]
Kidney disease	Bushen Huoxue formula	[81]
Leukemia	Realgar-Indigo naturalis formula	[266]
Liver disease	Jian Gan Bao	[267]
	Zhi Zi Da Huang decoction	[268]
Lung cancer	Xia Qi Decoction	[269]
	Fuzheng Kangai	[215]
	kushen injection	[216]
Maintain the stasis of blood	Danggui	[270]
	Buyang Huanwu decoction	[271]
Migraine	Da Chuanxiong formula	[272]
Myocardial infarction	Xuesaitong injection	[273]
	QiShen YiQi	[274]
	Shenmai injection	[44]
Neurodegenratve diseases	Tinospora sinensis	[231]
	*Brucea javanica, Dichroa febrifuga, E. micranthum Harms, Erythrophleum guineense, Holarrhena antidysenterica,* and *Japanese ardisia*	[233]
	Shaoyao Gancao	[232]
	*Medicago sativa*	[234]
Osteoarthritis	Taohong Siwu decoction	[275]
Rheumatoid arthritis	Qing-Luo-Yin	[45]
	*Fructus schisandrae*	[276]
Thrombosis	Fufang Xueshuantong	[277]
Traumatic injury	Diesun Miaofang	[278]
Type 2 diabetes mellitus	Ge Gen Qin Lian decoction	[94]
